# Fecal strains *Enterococcus mundtii* from wild ruminants, their safety and postbiotic potential

**DOI:** 10.1007/s11259-025-10701-3

**Published:** 2025-03-19

**Authors:** Andrea Lauková, Valentína Focková, Marián Maďar, Grzegorz Belzecki, Renata Miltko, Monika Pogány Simonová

**Affiliations:** 1 Centre of Biosciences of the Slovak Academy of Sciences, Institute of Animal Physiology, Šoltésovej 4-6, 040 01 Košice, Slovakia; 2 Department of Microbiology and Immunology, University of Veterinary Medicine and Pharmacy in Košice, Komenského 73, 041 81 Košice, Slovakia; 3 The Kielanowski Institute of Animal Physiology and Nutrition, Polish Academy of Sciences, Instytucka 3, 05 110 Jablonna, Poland

**Keywords:** Wild ruminants, Enterococci, Benefit, Properties, Characteristic

## Abstract

Roe and red deers represent inhabitants in European forests but also in agricultural areas. In general, microbiota can have a significant impact on health. E. g. the genus *Enterococcus* was detected in more or less high abundance percentage in feces of red deers. To preserve negative impact of some microbiota, beneficial autochthonous strains can be selected for this purpose. The aim of this study was to assess safety, postbiotic activity and character of the fecal strains *Enterococcus mundtii* from roe and red deers living in Poland to spread basic microbiology research in this field and to select application candidate. Taxonomy of seven *E. mundtii* was determined using MALDI-TOF mass spectrometry and 16S rRNA sequence analysis. The evaluation score responded mostly with secure genus identification/probable species identification. Strains showed identity up to 100% with the sequence MK414812.1 of *E. mundtii* in GenBank. They were deoxyribonuclease and gelatinase- negative, with low-grade biofilm formation (0.100 ± 0.31 to 0.181 ± 0.43). *E. mundtii* were susceptible to antibiotics without production of damaging enzymes. They were absent of virulence factors genes (*gelE*,* agg*,* esp*, *efaAfm*,* efaAfs*). Postbiotic potential of the bacteriocin substance produced by *E. mundtii* revealed inhibition of indicator strains growth up to 48%. The most active substance was from the strain EM 1/90/2; inhibitory activity against enterococci, staphylococci and listeriae up to 86% (up to 1600 AU mL) was noted. The substance from the strain EM 6/123/1 reached inhibitory activity up to 81% with activity up to 400 AU/mL. It remained active at -20 ˚C for one month. *E. mundtii* were assessed with no and/or low- grade virulence factor rate and with postbiotic potential for further studies.

## Introduction

Roe deer (*Capreolus capreolus*) is a common inhabitant in European forests (Wilson et al. 2019) but also in agricultural areas (Dahl et al. 2023). Red deer (*Cervus elaphus*) is also a typical forest-inhabiting mammal (Sun et al. 2023). Information associated with wild ruminants microbiota has been reported in literature especially regarding the gut/rumen microbiota which form a complex microecosystem in vertebrates and it has been affected by various factors (Sun et al. 2023). Dahl et al. (2023) reported that the bacterial microbiota in the rumen content of roe deer differed significantly in terms of habitat, season and age; but for example gender was not causing a significant difference in microbiota content. Microbiota can have even a significant impact on the health of the host, such as promoting immunity, digestion, and/or metabolism (Nicholson et al. 2012). Many studies have shown diet is an important factor affecting the structure and function of the fecal microbiota as well as the relative abundance of some bacteria (Zhang et al. 2010).

The phylum Firmicutes was found as the most frequent phylum in the roe deer rumen content (61%), followed by Bacteroidota (19%), and Actinobacteroita (14%). Dahl et al. (2023) found 277 different genera with five core microbiota such as *Christensenecellacae*, *Prevotella*,* Oscillospiracae*, *Eggerthellaceae*, and *Lachnospiracae*. In additon to the core microbiota, other genera were determined with higher abundance such as *Fretibacterium*,* Latilactobacillus*,* Syntrophococcus*,* Streptococcus*,* Lentilactobacillus*,* Ralstonia*,* Tyzzerella*,* Catenisphaera*,* Enterococcus* and *Leuconostoc* (Dahl et al. 2023). In red deer (*Cervus elaphus*) 10 top fecal phyla (using next-generation sequencing) have been already indicated such as dominated phyla Firmicutes, Bacteroidetes, Tenericutes, Actinobacteria, Proteobacteria, Spirochaetes, Cyanobacteria, TM7, Verrumicrobia, Acidobacteria (Sun et al. 2023) and 30 top genera. What it is interesting that in case of rumen content but also in case of red deer feces, the genus *Enterococcus* was indicated in more or less high abundance percentage. So, it means that microbiota can be influenced by different factors through beneficial and/or negative direction. To preserve negative direction, autochthonous strains can be studied to have beneficial, mostly postbiotic character. Because one way is to know microbial taxonomy in detail but second way is to have in hand real strain which could be beneficially used.

Enterococci have evolved as vastly amended members of the intestinal microbiota of a wide range of hosts (Nawaz et al. 2019). In the past, enterococci have been considered as opportunistic pathogens due to antibiotic resistance genes presence or other virulence factor genes detection. However, nowadays, more studies suggested the role of specific enterococci as beneficial strains for animal and also human use (Franz et al. 2011; Pogány Simonová et al. 2020). The species *Enterococcus mundtii* is assigned as a member of group *E. faecium* on the basis of homology in 16S rDNA sequence (Nawaz et al. 2019). The GC content of this bacterium ranges between 38 and 39%. The representants of this species can produce enterocins (antimicrobial proteinaceous substances- postbiotics), which can be used to prevent mastitis (Espeche et al. 2009). Postbiotics are defined as preparations of inanimate microorganisms and/or their components that confer a health benefit to the host (Salminen et al. 2021).

Therefore, the aim of this study was to assess postbiotic activity and character of enterococcal strains (*E. mundtii*) isolated from roe and red deers living in Poland. The strains were taxonomically identified using MALDI-TOF MS spectrometry (Matrix-Assissted Laser Desorption/Ionization Time-of-Flight Mass Spectrometry) and also using sequencing method. MALDI-TOF MS spectrometry is a method recommended especially for research and/or clinical microbiology. It is also indicated as revolutionized procedure for bacteria indetification (Paskova et al. 2020). The 16S rRNA gene sequencing is an amplicon-based sequencing method that is used to identify and classify bacteria (Muhammad Rizal et al. 2020). Moreover, the aim of this study was also denoted to spread basic microbiology research associated with wild animals and possible application of selected candidate to sustain One Health concept.

## Materials and methods

### Sample processing

The feces of wild ruminants (21 free-living, 12 young female roe deers and 9 adult female red deers) were sampled in Poland (the Strzalowo Forest District, Piska Primaveal Forrest 53˚ 36 × 43.56“ N, 21˚ 30 × 58.68“ E) by our Polish colleagues with whose we co-operated as previously described by Lauková et al. (2020a). The animals were killed by selective-reductive shooting during December/January of the years 2014/2015 according to annually planned hunting approved by the proper district forester of the state forests in agreement with Polish Hunting Association. After the animals were shot, each sample in the field was labelled by marking the date, location and the age and sex of specimen as well. At that time, stored samples (4 ˚C) were transported to our laboratory by a Courier company. First (after 24 h), the samples were treated by the standard microbiological method according to ISO (International Organization for Standardization); one g of each fecal sample was diluted in Ringer solution (pH 7, Merck, Darmstadt, Germany, ratio 1:9). The appropriate dilutions were spread on M-Enterococcus agar (Difco, USA) and incubated at 37 ˚C for 48 h. Grown colonies were picked up and maintained on Brain-heart agar (BHA, Difco, USA) supplemented with 5% of defibrinated sheep blood for next identification.

### Species taxonomy identification: MALDI-TOF mass spectrometry

Each one picked up colony (21) from BHA agar enriched with blood was submitted for MALDI-TOF mass spectrometry identification (Bruker Daltonic 2011) as previously described by Lauková et al. (2020a). Prior to identification, lysates of bacterial cells were prepared according to manufacturers instructions (Bruker Daltonic 2011). Mass spectrometry based on the MALDI-TOF means that the bacterial isolate to be analyzed is adsorbed to some type of matrix (carrier material). Then it is irradiated with laser UV light, so that the molecules in the bacteria are broken into positively charged fragments (ionization) which are thrown towards a detector. The time takes for the fragment to reach the detector (time of flight) is measured and it is dependent on fragment size and charge. Large molecules (proteins, nucleic acids) give rise to many fragments and a characteristic mass spectrum which can be used for identification (Fedorko et al. 2012). Evaluation score classified strains with secure genus identification/probable species identification ranging from 2.000 to 2.299, then 2.300–3.000 indicating high probable species identification and 1.700–1.999 probable genus identification. As positive controls served reference strains included in the identification system.

### Species taxonomy identification: DNA extraction, PCR amplification, sequence analysis

Detail description of the method was reported previously by Focková et al. (2022a). DNAzol direct (Molecular Research Centre Inc. Cincinnati, USA) was used to extract the genomic DNA from a pure solitary colony. The genus and species were determined by BLASTn (basic local search tool) homology analysis (http://blast.ncbi.nlm.nih.gov/blast.cgi) of the 16S rRNA gene sequence which was amplified using the universal primers Bac27F (5´-AGAGTTTGATCCTGGCTCAG-3´) and 1492R (5´-CGGTTACCTTGTTACGACTT-3´, Merck-Sigma Aldrich, Darmstadt, Germany). Amplified products were sent for purification in low bind tube at minimal volume 15 µL and for sequencing in both directions using formerly indicated primers. The PCR mixture (50 µL) per sample contained 2 µL of DNA shield, 46 µL of a reaction mixture comprising One Taq 2x Master Mix with Standard buffer (New England Biolabs, United Kingdom), diluted with water for molecular biology (PanReac AppliChem, Darmstadt, Germany) to 1 x concentration and 1 µL of each primer (33 µM). The reactions were performed on TProfesional Basic thermocycler, Biometra GmbH, Goettingen, Germany) under these conditions: 5 min at 94 ˚C, followed by 30 cycles at 94˚C for 1 min, annealing at 55 ˚C for 1 min, then at 72 ˚C for 3 min, and a final extension step at 72˚C for 10 min. Aliquot PCR products were analyzed on 3% (w/v) agarose gel electrophoresis in Tris-acetate-EDTA buffer (pH 7.8). They were visualized with GelRed (Biotium Inc. Hayward, CA, USA). The 16S rRNA sequences were validated and assembled using Geneious 8.05 (Biomatters, Auckland, New Zealand) and subjected to BLASTn analysis as indicated. This method uses the highly conserved nature of the 16S ribosomal RNA (rRNA) gene present in all prokaryotes that also contains variable regions that differentiate between species. The development of 16S rRNA as markers for the identification of organisms along with the development of next-generation sequencing (NGS) techniques has allowed efficient and rapid study of numerous samples (Sanju Tamang 2024).

### Enzyme activity measurement (API ZYM set)

The API ZYM panel system (BioMeriux, Marcy l Etoile, France) was applied to test enzyme activity of seven identified *E. mundtii* as previously described Focková et al. (2022b). An amount of 65 µL inoculum (McFarland standard) was transferred into each one well of the panel system kit. After incubation at 37 ˚C for 4 h, the appropriate reagents ZYM A and ZYM B were added. The enzyme activity was evaluated by color intensity values checking (from 0 to 5). Their relevant value in nanomoles (nmoL) were assigned for each reaction according to chart supplied with the test. The panel test involves 19 enzymes such as alkaline phosphatase, esterase (C4), esterase lipase (C8), lipase (C14), leucine arylamidase, valine arylamidase, cystine arylamidase, trypsin, α-chymotrypsin, acid phosphatase, naphtol-AS-BI-phosphohydrolase, α-galactosidase, β-galactosidase, β-glucuronidase, α-glucosidase, β-glucosidase, N-acetyl-β-glucosaminidase, α-mannosidase, and α-fucosidase.

### Antimicrobial (antibiotic) phenotypic status

The disc diffusion method was used to investigate the antibiotic susceptibility and/or resistance features of *E. mundtii* strains (CLSI 2016). The commercial antibiotic discs obtained from Oxoid Ltd. (Basingstoke, England) were used in the disc diffusion test: novobiocin, streptomycin (5 µg), penicillin G (10IU), ampicillin (10 µg), erythromycin, azithromycin (15 µg), chloramphenicol, kanamycin, tetracycline, rifampicin, and vancomycin (30 µg). Broth cultures of tested strains (100 µL) were spread onto Mueller-Hinton (Difco) agar with blood. Then antibiotic discs were applied. The plates were incubated overnight at 37 ˚C. The inhibitory zones were evaluated and expressed in mm and reported according to the guidelines of the Clinical and Laboratory Standards Institute (2016). *E. mundtii* EM 2/2 (Lauková et al. 2020c) served as positive control.

### Biofilm-forming ability analysis

The quantitative plate assay was applied to test biofilm-forming ability of identified *E. mundtii* strains according to Chaieb et al. (2007) and as previously described by Focková et al. (2022b). Tested strain (one colony) grown on Brain heart agar overnight (Difco, Maryland, USA) was transferred into 5 mL of Ringer solution (pH 7, Merck, Darmsdadt, Germany) to obtain 1 × 10^8^ cfu/mL. Then a 100 µL aliquot from that suspension was transferred into 10 mL of Brain heart infusion (BHI). A 200 µL aliquot of this dilution was inoculated into mcrotiter plate wells (Greiner ELISA 12 Well Strips, 350 µL flat bottom Frickenhausen GmbH, Germany) and incubated at 37 ˚C for 24 h. The forming biofilm in the microtiter plate wells was washed twice with 200 µL of deionized water and dried at 25 ˚C for half hour. The remaining bacteria were stained for half hour at 25 ˚C using 200 µL of 0.1% (w/v) crystal violet in deionized water. After dye solution was aspirated away, the wells were washed twice with 200 µL of deionized water. The plate was dried (30 min at 25 ˚C) and the dye bound to the adhered biofilm was extracted with 200 µL of 95% ethanol and stirred. Next, a 150 µL aliquot was transferred into new microplate well to measure absorbance (A) at 570 nm (Synergy TM4 Muldi Mode Microplate reader, Biotek USA). Each strain and condition was analyzed in two independent analyses with 12 replicates. A sterile BHI medium was included in each testing as a negative control. *Streptococcus equi* subsp. *zooepidemicus* CCM7316 served as a positive control (provided by Dr. Styková from University of Veterinary Medicine and Pharmacy in Košice, Slovakia). Biofilm formation was evaluated as highly positive (A_570_ ≥ 1), low-grade positive (0.1 ≤ A_570_ **<** 1) or negative (A_570_ **<** 0.1) as reported Chaieb et al. (2007).

### Safety assessment: hemolysis, nuclease and gelatinase activity demonstration

Hemolysis was controlled by streaking the strains onto BH agar (Difco, USA) supplemented with 5% defibrinated sheep blood. After incubation at 37˚ C overnight, the presence/absence of cleared zones around the colonies was interpreted as α, β- hemolysis and negative strains exhibited ɤ-hemolysis (Semedo-Lemsaddek et al. 2003).

Nuclease activity was tested as previously described by Lauková et al. (2020a). The tested strain was inoculated onto the surface of DNase agar (Oxoid, USA). After incubation at 37 ^˚^C for 24 h, the production of deoxyribonuclease can be evaluated as colonies producing DNase hydrolyse the DNA within the medium. After flooding with HCl, the DNA precipitated which became medium turbid with cleared zone around DNA-positive colonies. *Staphylococcus pseudintermedius* SPs 948 (our strain) served as positive control.

Gelatinase phenotype was tested by streaking single colonies onto BH agar (Difco, USA) supplemeted with gelatine 30 g/L- BioMark, Focková et al. 2022a). The plates were incubated at 37 ˚C overnight. After flooding the medium with HgCl_2_ (1.5%) in 2.0% HCl, the medium became turbid with cleared zones around gelatinase-positive colonies. *S. aureus* ATCC 25923 was used as positive control.

### In vitro safety assessment: virulence factor genes determination

In this test, the presence of the following virulence factor genes was analyzed as previously described by Kubašová et al. (2017): *gelE* (gelatinase), *esp* (enterococcal surface protein), *agg* (aggregation substance), *efaAfs* (adhesin *Enterococcus faecalis*), and e*faAfm* (adhesin *E. faecium).* The DNA from the strains was extracted using the rapid alkaline lysis (Baelae et al. 2001). The PCR method was performed in 25 µL volume; mix consisted of 1 x reaction buffer, 0.2 mmol/L of deoxynucleoside triphosphate, 3 mmol/L MgCl_2_, 1 µmol/L of each primer, 1U of Taq DNA polymerase, and 1.5 µL of DNA template. The following cycling conditions were used: initial step of 95 ˚C for 3 min, 35 cycles of 30 s at 95 ˚C, 30 s at 55˚ C, 30 s at 72 ˚C, and a final step at 72˚ C for 5 min. The PCR products were separated by agarose gel electrophoresis (1.2% w/v, Sigma-Aldrich, Saint Louis, USA) containing 1 µL/mL ethidium bromide (Sigma-Aldrich) using 0.5 x TAE buffer (Merck, Darmstadt, Germany). The PCR fragments were visualized with UV light. The positive control strains were *E. faecalis* P36 and *E. faecium* F10 (kindly provided by Dr. Teresa Semedo-Lemsaddek, University of Lisbon, Portugal).

### Beneficial postbiotic potential

At first, postbiotic (bacteriocin-antimicrobial activity) potential of *E. mundtii* strains was checked using the qualitative method. Bacteriocin (postbiotic) active strains were inoculated onto Brain heart agar in a line and incubated at 37 ˚C overnight. The indicator bacteria were grown overnight in appropriate broth according to the species. Their absorbance (A_600_) was measured and the volume 200 µL was mixed with 0.7% (w/v) agar. The agar with grown producing strain was overlaid with the mixture. After incubation (37 ˚C overnight) cleared zone around producing strain line was assessed as inhibition of indicator strain and expressed in size mm (zone in average more than 10 mm). Eighty-nine (89) indicator species strains were used: 42 different enterococci (*Enterococcus durans*,* E. casseliflavus*,* E. faecium*,* E. faecalis*,* E. mundtii*,* E. thailandicus*,* E. moraviensis*,* E. saccharolyticus)*, 25 staphylococci (*S. aureus*,* S. warneri*,* S. sciuri*,* S. vitulinus*,* S. epidermidis*,* S. haemolyticus*,* S. pseudintermedius*,* S. capitis*,* S. sciuri*,* S. hominis*), 9 *Streptococcus gallolyticus*, and 10 Gram-negative *E. coli*. Indicator strains were isolated from horses, beaver, ostriches, sportive mouthgards, rumen (our strains, EMo1-Nik 1 and *E. saccharolyticus* kindly supplied by Dr. Styková University of Veterinary Medicine and Pharmacy in Košice, Slovakia), also from Czech Culture Collection of Microorganisms in Brno, Czech Republic -CCM and/or University of Ghent). *E. avium* EA5 is the principal indicator. In addition, three strains of *Listeria monocytogenes* (Czech Veterinary Administration, Olomouc, Czech Republic) were tested.

After the qualitative test, cell-free supernatants were tested and then also concentrated supernatant against the strain EA5 using agar spot test according to De Vuyst et al. (1996). The supernatants were obtained by centrifuging broth cultures of the strains (10 000 x g for 30 min grown up to A_600_ 1.0 and pH 5.8–6.0, Jouan MR18, France). Supernatants were tretaed with EDTA and heated for 10 min at 80 ˚C. Then they were checked using agar spot test and inhibitory activity was expressed in arbitrary unit per mL (AU/mL). It means two-fold dilution of supernatant -bacteriocin which can cause inhibition of indicator strain). Dilutions were performed in phosphate buffer pH 5.5.

Concentrated bacteriocin substance was prepared after overnight growth of *E. mundtii* strains in BHI (18 mL). Then, the broth culture was centrifuged, and treated as described formerly. Next step was concentration using Concentrator plus (Eppendorf, Spain) at 30 ˚C for 4 h to reach 4 fold concentrated substance-concentrate. Then again the inhibitory activity was checked using the method according to De Vuyst et al. (1996).

Stability of supernatants, concentrates was checked after one month storage at −20˚C using agar spot test with *E. avium* EA5 as the principal indicator (the most sensitive indicator).

## Results

### Strains taxonomy

Using MALDI-TOF mass spectrometry, seven strains were allotted to the species *Enterococcus mundtii* (Table 1); six identified fecal *E. mundtii* were isolated from roe deers and only one strain *E. mundtii* was isolated from feces of red deer. The evaluated score of the strains EM 1/90/2, EM 1/133/1 and EM 5/114/1 responded with secure genus identification/probable species identification (2.000–2.299). Four strains (EM 3/166/1, EM 4/112/1, EM 6/123/1 from roe deer and EM 5/107/2 from red deer) were evaluated with score responded to probable genus identification (1.700–1.999). However, the species allotment was also identified using next-generation sequencing method (BLASTn analysis) by the species allotment with high percentage identity 16S rRNA sequence in all strains up to 100% (Table 1). Five out of seven strains reached even 100% identity and in two strains (Table 1) also high % identity (99.1, 99.91% respectively) was noted with the sequence MK414812.1of *E. mundtii* in GenBank.

**Table 1:** Fecal strains *Enterococcus mundtii* from roe and red deers (sampled in Poland) with MALDI-TOF mass spectrometry identification score, the identity percentage (BLASTn 16S rRNA sequence) and biofilm-forming ability

Strain	MALDI-TOF MS score	% identity Blastn	Biofilm
		16S rRNA seq.	
roe deer			
EM1/90/2	2.022	100%	0.181 ± 0.43
EM1/133/1	2.131	99. 1%	0.121 ± 0.34
EM3/166/1	1.801	100%	0.107 ± 0.32
EM4/112/1	1.912	99.91%	0.106 ± 0.32
EM5/114/1	2.019	100%	0.115 ± 0.33
EM6/123/1	1.995	100%	0.089 ± 0.00
red deer			
EM5/107/2	1.979	100%	0.100 ± 0.31

EM-*Enterococcus mundtii*; *E. mundtii* from database Brucker Daltonics, Billerica, Maryland, USA); sequence match in GenBank *E. mundtii* (MK414812.1);seq.-sequence

### In vitro safety assessment

The strains were deoxyribonuclease (DNase)-negative. In six strains was revealed β-hemolytic activity and EM 3/166/1 showed α-hemolytic activity. Low-grade biofilm -forming ability was measured in six strains (Table 1) with values from 0.100 ± 0.31 -EM 5/107/2 to 0.181 ± 0.43- EM 1/90/2. The strain EM 6/123/1 did not form biofilm. However, bacteriocin substance from this strain was active (with inhibitory postbiotic potential) even after one month storage at −20˚C.

The disc diffusion test revealed that *E. mundtii* strains were susceptible to ampicillin (inhibitory zones size 11–20 mm), penicillin G (16–24 mm), chloramphenicol (20–24 mm), erythromycin (19–23 mm), azithromycin (16–23 mm), vancomycin (15–18 mm), novobiocin (16–20 mm), rifampicin (22–31 mm), and tetracycline (24–30 mm). It means that the strains were susceptible to 9 out of 11 antibiotics tested. The strains showed resistance only to two antibiotics: streptomycin and kanamycin. Although kanamycin in relation with enterococci, could be chromozomally encoded, similarly as streptomycin. In CLSI, it is not requested for enterococcal antibiotic profile testing.

Production of damaging enzymes such as trypsin, α-chymotrypsin and/or β-glucuronidase was not detected in *E. mundtii* strains. The alkaline phosphatase was produced by only EM 1/90/2 strain in amount 5 nmoL. Esterase was measured in all strains (5 nmoL and 10 nmoL in strains EM 1/133/1 and EM 1/90/2). Also production of esterase lipase was detected in all strains (5–10 nmoL). Leucin arylamidase was detected in six strains (except EM 6/123/1). Moreover, *E. mundtii* strains did not produce lipase, valine arylamidase, α-mannosidase, α-fucosidase, and N-acetyl-β-glucosaminidase as well. Acid phosphatase was measured in two strains (EM 4/112/1 and EM 3/166/1), similarly as cystine arylamidase (EM 1/133/1 and EM 3/166/1). Production of naftol-AS-BI-phosphohydrolase was measured in all strains (5 nmoL). The strains did not produce also α-galactosidase, and α-glucosidase. The enzyme β-glucosidase was produced by four strains (EM 5/114/1 EM 1/133/1, EM 4/112/1 and EM 3/166/1). The useful enzyme β- galactosidase was produced only by the strain EM 4/112/1 (5 nmoL).

The strains *E. mundtii* lacked tested virulence factor genes (*gelE*,* agg*,* esp*, *efaAfm*,* efaAfs*). Moreover, none of the strains exhibited gelatinase activity phenotype.

### Beneficial postbiotic potential

The substance produced by the strain *E. mundtii* EM 1/90/2 inhibited the growth 36 out of 42 enterococci (up to 86%) but only six strains out of 25 staphylococci (24%). Among listeriae one strain out of three tested was inhibited. The indicator strains *Str. gallolyticus* and Gram-negative *E. coli* were not inhibited by this and also by the other tested strains (Table 2). Twenty-nine out of 42 (69%) enterococcal strains were inhibited using substance produced by the strain EM 1/133/1 and 28% (7) staphylococci. Again the growth of one strain *L. monocytogenes* LM7223 was inhibited. In case of the substance from the strain EM 3/166/1, 31 out of 42 enterococcal indicators were inhibited (73.8%) followed by 5 staphylococci out of 25 (20%) and one *L. monocytogenes*. The substance from the EM 4/112/1 inhibited the growth of 29 enterococci (69%), 20% staphylococci (5 out of 25) and one *L. monocytogenes*. The substance from the strain EM 6/123/1 inhibited the growth of 34 out of 42 enterococci (81%) and 4 out of 25 (16%) staphylococci and one strain *L. monocytogenes*. The least active was substance produced by the strain EM 5/114/1 which inhibited almost 5% of enterococci (2 out of 42) and 2 out of 25 (8%) staphylococci. Similarly the least active was substance from red deers EM 5/107/2 with growth inhibition only one *E. avium* strain (EA5) and none one staphylococcal strain was inhibited and any *L. monocytogenes* strain as well. In total, postbiotic active substance from the strain EM 1/90/2 inhibited the growth of 48% indicators, followed with the substance EM 1/133/1 reaching 42% of inhibited indicators. The same percentage (42%) was reached by the substance EM 3/166/1. Using the substance EM 4/112/1, 39% indicators were inhibited in total. The substance from EM 6/123/1 inhibited 43% of indicator strains in total; however, this substance showed stable inhibitory activity even after one month storage at −20˚ C (Table 3). Oppositelly, the substance from the strain EM 5/114/1 inhibited only 5% and from EM 5/107/1 only 1% indicators. It means, the highest postbiotic potential showed substance EM 1/90/2, followed by the substance EM 6/123/1 and the others except EM 5/114/1 and EM 5/107/2.

**Table 2:** Postbiotic potential (number of inhibited strains) due to bacteriocin activity

	1/90/2	1/133/1	3/166/1	4/112/1	5/114/1	6/123/1	5/107/2
*E. avium*	1/1	1/1	1/1	1/1	1/1	1/1	1/1
*E.thail.*	8/8	8/8	8/7	8/8	8/1	8/8	8/0
*E. durans*	4/4	4/4	4/4	4/4	4/0	4/4	4/0
*E. cassel.*	2/1	2/1	2/1	2/1	2/0	2/1	2/0
*E. mor.*	1/1	1/1	1/1	1/1	1/0	1/1	1/0
*E. sach.*	1/1	1/1	1/1	1/1	1/0	1/1	1/0
*E. faecium*	7/7	7/7	7/7	7/7	7/0	7/7	7/0
*E. mundtii*	17/13	17/6	17/9	17/6	17/0	17/11	17/0
*S.aureus*	6/1	6/2	6/2	6/1	6/0	6/1	6/0
*S. haem*.	4/1	4/1	4/0	4/0	4/0	4/1	4/0
*S.warneri*	5/2	5/2	5/1	5/2	5/2	5/0	5/0
*S.sciuri*	2/1	2/1	2/1	2/1	2/0	2/1	2/0
*S. vit.*	1/1	1/1	1/1	1/1	1/0	1/1	1/0
*L. mon*.	3/1	3/1	3/1	3/1	3/0	3/1	3/0

x/x: number of the strains tested/number of the strains inhibited; Ruminant-*S. pseudintermedius* SPs948 (1/0), canine *S. epidermidis* 5 Pa (10), canine-*S. hominis* (3/0), *S. saprophyticus* (1/0), *S. capitis* (1/0), *S. vitulinus*, beaver-*Streptococcus gallolyticus* (9/0), horses-*Escherichia coli* (10/0); clinical strains *Listeria monocytogenes*, *E. avium* EA5-piglet (our strain), beaver-*E. thailandicus* (8), E. *moraviensis*,* E. saccharolyticus* (1x horses), 17 fecal horses *E. mundtii*,* 7 E. faecium*, *E. casseliflavus* 2, 1 x clinical strain -*E. faecalis* CCM4224 (1/0), size of inhibitory zones ranged from 9 to 24 mm); In case of *E. avium* EA5 the inhibitory zone reached 24 mm

**Table 3:** Postbiotic activity of *E. mundtii* supernatants and concentrated substances after one month storage at −20 ˚C tested against *Enterococcus avium* EA5 (in arbitrary unit per milliliter-AU/mL)

Strains	Supernatant	Concentrate	Month (−20˚C)
EM1/90/2	100	1 600	0
EM1/133/1	100	800	0
EM3/166/1	100	800	0
EM4/112/1	100	400	0
EM5/114/1	100	400	0
EM6/123/1	100	400	100
EM5/107/2	0	0	0

The most postbiotic active substance (EM 1/90/2) also reached the highest inhibitory activity testing by the quantitative method (1 600 AU/mL, Table 3); however, this substance was not stable at −20 ˚C stored for one month. On the other hand, the substance with postbiotic activity 400 AU/mL (from the strain EM 6/123/1) remained active also after one month storage at −20 ˚C (Table 3).

## Discussion

The taxonomy of identified *E. mundtii* strains was confirmed twice, using MALDI-TOF mass spectrometry and BLASTn 16S rRNA sequencing. Nowadays, these methods are the most frequently recommended and validated for bacteria identification. These methods were also applied in previous studies for taxonomic allotment of fecal enterococci e.g. from horses (Focková et al. 2022a) and other enterococci as well (Lauková et al. 2020b). Moreover, the additional properties tested contribute to more appropriate characteristics of studied strains and to their complex characterization.

The enzyme activity represents a parameter evaluated in strains for both beneficial but also damaging enzymes. In general, there are some bacterial groups such as clostridia, staphylococci, coliforms, etc., the representatives of which are frequent producers of β-glucuronidase, the enzyme related to diseases (Chamseddine et al. 2019), cancer including. Focková et al. (2022b) reported fecal horses strains *E. mundtii* with higher production of metabolic enzymes comparing with those from wild ruminants (roe and red deers); however, production of the enzymes such as β-glucuronidase, trypsin and α-chymotrypsin was not detected. For example, alkaline phosphatase is an enzyme playing role in metabolism within liver and skeleton and it works as an marker for hepatitis diagnosis. So no detection of those type enzymes production in tested bacteria indicated that they are not associated with diseases agents. On the other side, in fecal *E. mundtii* from horses, useful enzyme β-galactosidase production was measured (Focková et al. 2022b). This enzyme is active in the mucosa of the small intestine.

The PCR test showed that tested *E. mundtii* did not contain the virulence factor genes. Similar to our findings, Genis et al. (2024) also confirmed absence of virulence factor genes in animal colostrum-derived species strains *E. mundtii.* But Genis et al. (2024) reported β-hemolytic and also weak α-hemolytic activity in the species strains *E. mundtii* isolated from sheep and goats colostrum. Hemolytic activity plays a significant role in infections. However, another strain *E. mundtii* EM 2/2 (isolated from raw goat milk) exhibited ɤ-hemolysis meaning it was hemolysis- negative. Due to the safety concerns, β-hemolytic strains should not be used as adjunct in food. But considering hemolytic featers, some probiotic strains in food, *E. faecalis* or *E. faecium* have higher levels of β-hemolytic activity (Eaton and Gasson 2001). Similarly as in our study presented fecal strains *E. mundtii*, also milk-derived strain *E. mundtii* EM 2/2 was gelatinase, DNase- negative and did not form biofilm (Lauková et al. 2020c). Moreover, Focková et al. (2022a) presented fecal strains *E. mundtii* from Slovak horse breed Norik from Muráň which were hemolysis-negative (ɤ-hemolysis), DNase and gelatinase- negative with antibiotic susceptibility referring with that reported here.

Antibiotic resistance is a part of selecting whether enterococcal strains are safe or not (Zommiti et al. 2018). Because strains can be potential reservoir for antibiotic resistance genes. In our study tested strains showed mostly susceptible antibiotic phenotype which is usually in correspondance with no resistance genes detection. Moreover, *E. mundtii* were susceptible to the glycopeptide -group of antibiotics such as e.g. vancomycin what is advantageous fact (Zommiti et al. 2018). The benefit of susceptibility to vancomycin in tested *E. mundtii* strains lies in the fact that an increase of vancomycin-resistant enterococci (VRE) have been noted over world. They have emerged as non-requested agents especially regarding the clinical isolates (Bonten at al. 2001). Similar antibiotic feature as in our study reported also Genis et al. (2024) for *E. mundtii* strains. In addition, *E. mundtii* EM 2/2, our strain isolated from raw goat milk showed susceptibility to clinically-important antibiotics and resistance to kanamycin and streptomycin which represents in most enterococcal species chromozomally coding marker (Lauková et al. 2020a). However, as formerly mentioned, *E. mundtii* EM 2/2 exhibited ɤ-hemolysis meaning it was hemolysis- negative.

In this study, low-grade biofilm formation was measured in six strains. A biofilm is a compacted assemblage of microorganisms enclosed in a matrix primarly composed of polysaccharide and attached on a surface (Sahin 2019). Bacteria grown in the form of a biofilm are characterized by increased resistance to host defense responses as well as natural resistance to antibiotic activity (Costerton 1999). In this case, *E. mundtii* were mostly susceptible to antibiotic activity and natural resistance to kanamycin, streptomycin was noted. Moreover, these strains produce bacteriocins. Molham et al. (2021) and Cotter et al. (2013) reported the use of bacteriocins for microbial biofilm control. Among *E. mundtii* strains in this study, six were found to produce biofilm and possess postbiotic potential against indicator strains. Surprisingly, that one substance produced by the strain EM6/123/1 which remained active even after one month storage at −20˚C was non-biofilm-forming. However, it is known, that biofilm-forming ability has been assessed as benefit in case of postbiotic active strains; on the other hand, this property is not essential for the strain which is postbiotic active. Focková et al. (2022b) also reported *E. mundtii* strains with low-grade biofilm-forming ability isolated from feces of horses. There even most of strains were not biofilm-forming. *E. mundtii* in this study were assessed with no and/or low -grade virulence factor rate. Nawaz et al. (2019) assessed *E. mundtii* QAUEM2808 isolated from artisanal fermented milk product Dahi as safe after virulence parameters evaluating and even using its application in mice. This strain was also found with a broad antimicrobial spectrum (postbiotic active) inhibiting Gram-positive and Gram-negative bacteria.

Oppositelly, bacteriocin (postbiotic) activity of the strain *E. mundtii* QU2 from soybean was directed only against relative bacteria (lactobacilli, enterococci) and also similarly as in our case representative of the genus *Listeria* was inhibited (*L. innocua* ATCC 33090^T^) with inhibitory activity 102 400 AU/mL (Zendo et al. 2005). Several bacteriocins produced by *E. mundtii* were reported with anti-listerial activity e.g. Mundticin KS (Kawamoto et al. 2002) produced by the grass silage strain *E. mundtii* NFRI 7393. Focková et al. (2022a) reported fecal horses *E. mundtii* with a narrow spectrum of postbiotic activity; however, there the growth of staphylococci was inhibited and similarly as in our study, Gram-negative *E. coli* were not inhibited.

Storage stability of postbiotic activity is an important parameter to study possible application use of bacteriocins from practical aspect. Ferreira et al. (2007) tested *E. mundtii* for bacteriocin activity and stability. Here, the substance from the strain EM6/123/1 was stable at −20 ˚C (inhibitory activity 100 AU/mL). Although testing of the susbtance storage character will be continued in our case, it is surprise. That it is as reported Ferreira et al. (2007), the temperature 4 ˚C was better for postbiotic activity remaining in *E. mundtii*. Simonová and Lauková (2007) reported Enterocin 2019 produced by the rabbit-derived strain *E. faecium* EF2019 = CCM7420 which remained stable activity (25 600 AU/mL) even for 3 months at −20 ˚C. Activity of postbiotics (bacteriocins) can be mostly influenced by the media from which it is extracted, storage condition and/or with inital pH (Espeche et al. 2009). Phumisantiphong et al. (2017) reported bacteriocin EF478 which remained active even after one year storage at −20 ˚C. This is a promissing result because − 20˚ C is commonly used temperature in refrigerating freeze box which to easier their application condition.

## Conclusion

Seven identified *E. mundtii* strains providing safe character because of virulence factor absence (meaning no and/or low -grade virulence factor rate) with low-grade biofilm- forming ability showed postbiotic potential of their bacteriocin substances revealing inhibition of indicator strains growth up to 48%. The most postbiotic active substance was produced by the strain EM 1/90/2. The substance produced by the strain EM 6/123/1 remained active even after one month storage at −20 ˚C. Postbiotic activity of these strains will be continued to study for their further application potential.

## Data Availability

No datasets were generated or analysed during the current study.
